# 2-Methoxyestradiol Attenuates Testosterone-Induced Benign Prostate Hyperplasia in Rats through Inhibition of HIF-1*α*/TGF-*β*/Smad2 Axis

**DOI:** 10.1155/2018/4389484

**Published:** 2018-08-01

**Authors:** Ashraf B. Abdel-Naim, Thikryat Neamatallah, Basma G. Eid, Ahmed Esmat, Abdulmohsin J. Alamoudi, Gamal S. Abd El-Aziz, Osama M. Ashour

**Affiliations:** ^1^Department of Pharmacology and Toxicology, Faculty of Pharmacy, King Abdulaziz University, Jeddah, Saudi Arabia; ^2^Department of Pharmacology and Toxicology, Faculty of Pharmacy, Ain Shams University, Cairo, Egypt; ^3^Department of Pharmacology, Faculty of Medicine, King Abdulaziz University, Jeddah, Saudi Arabia; ^4^Department of Anatomy, Faculty of Medicine, King Abdulaziz University, Jeddah, Saudi Arabia

## Abstract

Benign prostatic hyperplasia (BPH) is a common disorder in the male population. 2-Methoxyestradiol (2ME) is an end metabolite of estrogens with pleiotropic pharmacological properties. This study aimed to explore the potential ameliorative effects of 2ME against testosterone-induced BPH in rats. 2-Methoxyestradiol (50 and 100 mg/kg, dissolved in DMSO) prevented the rise in prostatic index and weight in comparison to testosterone-alone-treated animals for 2 weeks. Histological examination indicated that 2ME ameliorated pathological changes in prostate architecture. This was confirmed by the ability of 2ME to decrease the glandular epithelial height when compared to the testosterone group. Also, 2ME improved testosterone-induced oxidative stress as it inhibited the rise in lipid peroxide content and the exhaustion of superoxide dismutase (SOD) activity. The beneficial effects of 2ME against the development of BPH were substantiated by assessing proliferation markers, preventing the rise in cyclin D1 protein expression and enhancing Bax/Bcl2 mRNA ratio. It significantly reduced prostate content of tumor necrosis factor *α* (TNF-*α*), interleukin-1*β* (IL-1*β*), nuclear factor *κ*B (NF-*κ*B), and transforming growth factor *β* (TGF-*β*). In addition, 2ME reduced hypoxia-inducible factor 1-*α* (HIF-1*α*) and phospho-Smad2 (p-Smad2) protein expression compared to the testosterone group. In conclusion, 2ME attenuates experimentally induced BPH by testosterone in rats through, at least partly, inhibition of HIF-1*α*/TGF-*β*/Smad2 axis.

## 1. Introduction

Benign prostatic hyperplasia (BPH), which is characterized by increased proliferation of prostatic epithelial and stromal cells, is a common disorder in the male population [1]. It has been reported that the incidence of BPH increases with advancing age [2]. It is estimated that about a half of men aged 60 years experience symptoms of BPH. This number increases to 90% in men aged 85 years, as reported by the American Urological Association [3]. Prostatic tissue overgrowth is accompanied by a poor glandular elasticity that may lead to urethral opening constriction. Men suffering from BPH commonly exhibit a range of lower urinary tract symptoms (LUTS) including frequent and urgent urination, nocturia, urinary hesitancy, and a weakened urination. The molecular events leading to the development of BPH are not fully revealed. A number of overlapping risk factors have been suggested including ageing, oxidative stress, direct infection, urinary reflux, inflammation, and androgens which are somewhat intertwined [1, 4].

2-Methoxyestradiol (2ME) is a natural end metabolite of estrogens. It is produced by the oxidation of 17*β*-estradiol by the enzyme, cytochrome P450, and subsequently by O-methylation by catechol-O-methyltransferase (COMT). Yet, 2ME has a very low or almost no estrogenic activity [5]. Interestingly, 2ME has pleiotropic pharmacological actions including inhibition of tumor cell growth [6], metastasis [7], and angiogenesis [8]. In the same time, only trivial side effects were reported. The most frequent toxicities were fatigue, nausea, hypophosphatemia, cough, and abdominal bloating [9]. All trials have indicated that 2ME was well tolerated and exhibited excellent safety profile with no serious toxic effects [9–11].

Benign prostatic hyperplasia (BPH) patients show constant estradiol levels, whereas dihydrotestosterone declines with age. This suggests a role for estrogen and/or its metabolites in the pathogenesis of prostate disorders [12, 13]. 2-Methoxyestradiol selectively blocks hypoxia-inducible factor 1-alpha (HIF-1*α*) [14]. The latter has been implicated in the pathogenesis of BPH as it enhances androgen receptor signaling and together with tumor survival factors promotes prostate cell proliferation [15, 16]. HIF-1*α* and aromatic hydrocarbon (AH) receptor are components of a signaling network that supports resistance to cancer chemotherapy. AH receptors are degraded by the E3 ligase CHIP. Previous work demonstrated that 2ME induces phosphorylation of the E3 ligase CHIP and degradation of AH receptor and HIF-1*α* [17]. To the best of our knowledge, 2ME has not been tried yet against BPH. The aim of the present study was to examine the potential attenuating effects of 2ME in testosterone-induced BPH and elucidate underlying mechanisms.

## 2. Materials and Methods

### 2.1. Chemicals

2-Methoxyestradiol was purchased from Fraken Biochem Co. Ltd. (Qingdao, China) with purity more than 98%. Testosterone enanthate (STEROID S.p.A., Cologno Monzese, Italy) was provided by Chemical Industries Development Co. (CID), Giza, Egypt. Other chemicals were of the highest analytical grade available commercially.

### 2.2. Animals

All animal procedures were ethically approved by the Research Ethics Committee, Faculty of Pharmacy, King Abdulaziz University, number 1438-101. In addition, all methods were performed in accordance with US government guidelines for utilization and care of vertebrate animals used in testing, research, and training. Ten-week-old male Wistar rats (220–250 g) were obtained from King Fahd Medical Research Center, King Abdulaziz University. Rats were kept on a half-day light/dark cycle in air conditioning. A standard food pellet diet ad libitum and water were freely available to the animals. Animals' acclimatization was extended for one week before the study.

### 2.3. Experimental Design

Rats were randomly alienated into 4 groups (8 rats each) and treated 5 days/week, for 2 consecutive weeks. Group 1 (control group) was given 2.5 ml/kg DMSO (50%), the vehicle of 2ME IP and 1 ml/kg olive oil SC, and the vehicle of testosterone enanthate. Group 2 was given DMSO IP and 3 mg/kg testosterone SC for induction of BPH. Groups 3 and 4 were given 50 and 100 mg/kg 2ME (dissolved in 50% DMSO in a dosing volume of 2.5 ml/kg) IP, respectively, 1 h before testosterone enanthate administration. At 72 hours after the last testosterone dose, rats were sacrificed and the prostatic tissues were dissected out. Portions of the prostatic ventral lobes were kept in 10% neutral buffered formalin for histological and immunohistochemical processing. One-half of the remaining prostate tissues was stored for real-time polymerase chain reaction (RT-PCR) as provided in RNeasy Mini Kit (Qiagen, Hilden, Germany), and the other half was stored at −80°C for subsequent biochemical experiments.

### 2.4. Prostate Weight and Prostate Index

The prostate of each rat was rapidly dissected out and weighed. The prostate index was calculated for each rat by dividing the prostate weight by the body weight (mg/g).

### 2.5. Histopathology

Briefly, the fixed prostatic tissues from different groups were processed by paraffin technique to prepare sections of 4 *μ*m thickness. This was followed by deparaffinization, rehydration, and staining with hematoxylin and eosin (H&E). At least three of the stained sections were photographed and used to assess prostate glandular epithelial height using the image analysis software ImageJ, 1.46a, NIH, USA.

### 2.6. Determination of Markers of Oxidative Stress

Portions of the prostatic tissues were subjected to homogenization using ice-cooled phosphate-buffered saline (50 mM potassium phosphate, pH 7.5). The malondialdehyde (MDA) content was used to measure lipid peroxidation spectrophotometrically via the thiobarbituric acid reactive substance method, as previously described [18]. In addition, we determined the reduced glutathione (GSH) content and assessed the catalase (CAT) and superoxide dismutase (SOD) activities in prostate tissue homogenates using a commercially available kit (Biodiagnostic, Giza, Egypt). BCA protein assay kit (Biovision® Inc., CA, USA) was used to determine the protein content.

### 2.7. Cyclin D1 Detection by Immunohistochemistry

Tissue sections were dried using autoclave, deparaffinized, and rehydrated using ethanol. Then, they were boiled in citrate buffer (pH 6.0) for ten minutes. Afterwards, the sections were incubated in 5% bovine serum albumin (BSA) in tris-buffered saline (TBS) for two hours followed by immersion overnight at 4°C with the primary antibodies (1 *μ*g/ml), rabbit polyclonal anti-cyclin D1 antibody (ABCAM, Cambridge, UK). TBS was used to flush the slides which were then incubated with the biotinylated secondary antibody using Cell and Tissue Staining Kit (R&D Systems, MN, USA). Image analysis was performed for at least three sections per rat, recorded, and quantified by ImageJ analysis software (ImageJ, 1.46a, NIH, USA).

### 2.8. Analysis of Bax and Bcl-2 by Real-Time Polymerase Chain Reaction (RT-PCR)

#### 2.8.1. RNA Extraction and cDNA Synthesis

RNA extraction was carried out on prostatic tissues using the RNeasy mini kit (Qiagen) as per manufacturer's instructions. RNA integrity was checked by electrophoresis on 2% agarose gel. A NanoDrop was utilized to determine the purity and concentration of the extracted RNA. A SuperScript III first strand cDNA synthesis kit (Thermo Fisher Scientific) was used for cDNA synthesis in a 20 *μ*l reaction mixture as previously described [19].

#### 2.8.2. Primer Design

Primers were designed using Gene Runner software and synthesized by Macrogen (Korea). Primer sequence homology and total gene specificity were determined with BLAST analysis (http://www.ncbi.nlm.nih.gov/blast). Primers comprised sequences of different exons with spanning and flanking the introns, to avoid the amplification of genomic DNA (gDNA). Nucleotide sequences of the primers used are shown in Table 1.

#### 2.8.3. Quantitative RT-PCR

Relative expression of Bax and Bcl-2 genes was performed using 1 *μ*l synthesized cDNA (10 ng/*μ*l) as the template in 5 *μ*l PowerUp SYBR Green PCR Master Mix and 1 *μ*l each primer using 7500 Fast real-time PCR system (Applied Biosystems). The thermal cycle consisted of an initial uracil-DNA glycosylase activation of 2 min at 50°C, the DNA polymerase activation of 2 min at 95°C, followed by 40 cycles of 3 s at 95°C, and 30 s at 60°C. B2m was used as endogenous control gene, and triplicates were carried out for each data point. The specificity of qPCR reaction was confirmed by melt curve analysis. The quantification method selected to validate microarray results was the relative quantification (∆∆Ct) method [20]. Normalization was performed with the mean of B2m for the mean of the triplicate run per gene of interest.

### 2.9. Determination of IL 1*β*, TNF-*α*, TGF-*β*, and NF-*κ*B (p65)

Prostate homogenates were used to determine rat IL-1*β*, TNF-*α*, and TGF-*β* using ELISA kits according to the protocols provided by the manufacturers (ABCAM, Cambridge, UK). Prostate homogenates were used to prepare nuclear extracts using EpiQuik™ nuclear extraction kit (OP-0002, EpiGentek, NY, USA). Protein contents in the nuclear extracts were determined, and a fixed amount (100 *μ*g) was used in determining NF-*κ*B levels. Then, the nuclear fraction was used to assess NF-*κ*B using ABCAM's NF-*κ*B (p65) transcription factor assay kit (Cambridge, UK). Values of NF-*κ*B (p65) were expressed as fold change of the control group with reference of the recorded optical density.

### 2.10. Western Blot for HIF-*α* and p-Smad2 Protein Expression

Prostatic tissue lysate was prepared using ice-cold RIPA lysis buffer (ABCAM, Cambridge, MA, USA) supplemented with protease and phosphatase inhibitor cocktails, by sonicating the samples 5 times (10 seconds each time) on ice, keeping them in ice for half an hour, and followed by centrifugation for 10 minutes at 4°C and 14000 rpm. BCA protein assay kit (Biovision Inc., CA, USA) was used to determine supernatant protein. Protein samples (100 *μ*g) were loaded per well of SDS-PAGE gel (10% gel concentration) using electrophoresis buffer consisting of glycine (0.192 M), Tris (25 mM), and SDS (0.1%). Subsequent to electrophoresis, the gel was moved onto a PVDF membrane (Bio-Rad Laboratories, Hercules, CA, USA) using transfer buffer formed of glycine (0.192 M), Tris (25 mM), SDS (0.025%), and methanol (10%). Then, 5% milk in TBST was used to block the membrane followed by cutting into 2 pieces at molecular weight of 75 kDa. The membrane slice containing proteins higher than 75 kDa was incubated overnight with the primary rabbit polyclonal anti-HIF-1*α* antibody at 1 : 500 (sc-10790, Santa Cruz Biotechnology, CA, USA). Then, it was washed with 0.5% Tween-20 in Tris-buffered saline and incubated with HRP-conjugated anti-rabbit secondary antibody (1 : 5000). Thereafter, the membrane was developed by Enhanced Chemiluminescence (ECL) Western blotting kit (Licor®, NE, USA) and scanned on Licor C-Digit Blot Scanner (Model 3600, NE, USA). The other membrane slice (<75 kDa) was probed with rabbit monoclonal anti-phospho-Smad2 antibody at 1 : 1000 (ab-188334, ABCAM, Cambridge, UK). Subsequently, the slice was stripped, followed by reprobing with rabbit monoclonal anti-*β*-tubulin antibody (ab-179513, ABCAM, Cambridge, UK) as a loading control.

### 2.11. Statistical Analysis

Results are shown as mean ± SD. One-way ANOVA followed by Tukey as a post hoc test was used to conduct multiple comparisons to assess the statistical significance in the response between different groups. Differences between means were considered significant at *p* < 0.05. GraphPad Instat software version 3 was used for carrying out statistical analysis.

## 3. Results

### 3.1. Prostate Weight and Index

Primarily, 2ME was tolerated at all doses as treated animals showed no mortality or any signs of toxicity. Prostate weight and index were significantly increased by 76% and 93% in rats injected with testosterone enanthate, respectively, when compared to the control group (Table 2). Cotreatment with 2ME at the doses of 50 and 100 mg/kg caused a marked drop in the prostate weight by 34% and 52% and the prostate index by 18% and 47%, respectively, when compared to the testosterone-alone group. It was noted that treatment of rats with the used doses of 2ME for 2 weeks significantly decreased the body weight by 18% compared to control animals.

### 3.2. Histopathological Examination

**Figure 1:**
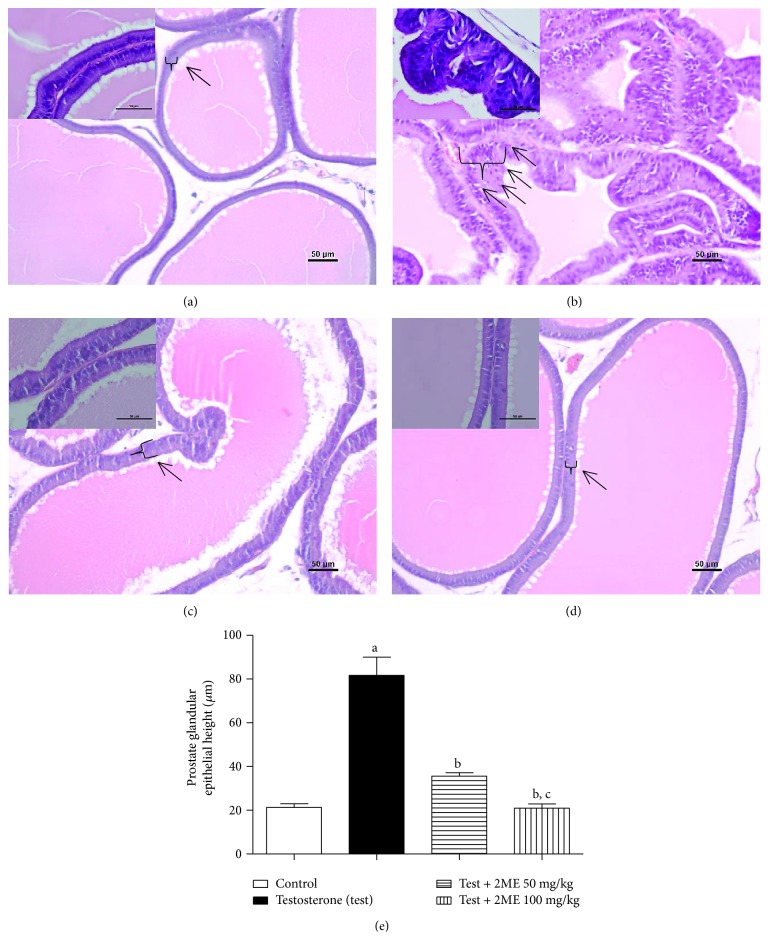
Histological examination of hematoxylin-eosin sections of rat prostates. (a) Section taken from control rats showing normal histoarchitecture of the ventral prostates. (b) Section taken from the ventral prostate of testosterone-treated group exhibiting hypertrophy with increased epithelial thickness and intraluminar projections. (c) Section taken from the ventral prostate of the testosterone group cotreated with 50 mg/kg 2ME showing mild reduction in hypertrophy and hyperplasia. (d) Section taken from the ventral prostates of testosterone groups cotreated with 100 mg/kg 2ME showing obvious reduction in prostate hypertrophy and hyperplasia. (e) A graph showing the effect of 2ME cotreatment on prostate glandular epithelial height. Scale bar (50 *μ*m). Data are expressed as mean ± SD (*n* = 6). a or b: statistically significant from control or testosterone group, respectively, at *P* < 0.05 using one-way ANOVA followed by Tukey's post hoc test.

Microscopic examination of H&E-stained sections of prostatic tissues taken from control rats showed normal structure in the form of numerous acini of different sizes and shapes containing homogenous acidophilic material. The lining epithelium of the acini was composed of two distinct cell types, namely, basal and acinar or principal cells. The acinar cells were columnar, extending from the basal lamina to the lumen of the duct. They were characterized by esinophilic granular cytoplasm and spherical or oval nuclei invariably located in the lower half of the cytoplasm (Figure 1(a)). Conversely, prostates obtained from testosterone enanthate-treated rats displayed an obvious disruption of the histoarchitecture of the prostatic tissue shown as marked hyperplasia, irregular acinar folding with intraluminar projections, and congestion (Figure 1(b)). 2-Methoxyestradiol at doses of 50 and 100 mg/kg was efficient in ameliorating hypertrophy, hyperplasia, and the intraluminar projections detected in the testosterone group, restoring almost normal histological structure (Figures 1(c) and 1(d)). These data were confirmed by assessing the height of glandular epithelia. 2-Methoxyestradiol significantly decreased the height by 56 and 74% at dose levels of 50 and 100 mg/kg, respectively (Figure 1(e)).

### 3.3. Oxidative Stress Markers

The data in Table 3 indicate that testosterone treatment caused a significant elevation of the lipid peroxidation marker MDA to almost 4 folds and decreased SOD activity by 30% compared to the corresponding control values. However, there were no significant changes in GSH content or CAT activity. Treatment with 2ME (50 and 100 mg/kg) significantly ameliorated testosterone-induced rise in lipid peroxidation by 30 and 59%. Also, 2ME significantly prevented SOD exhaustion and even normalized its values by the high dose.

### 3.4. Antiproliferative Activities of 2ME

#### 3.4.1. Assessment of the Proliferation Marker Cyclin D1

**Figure 2:**
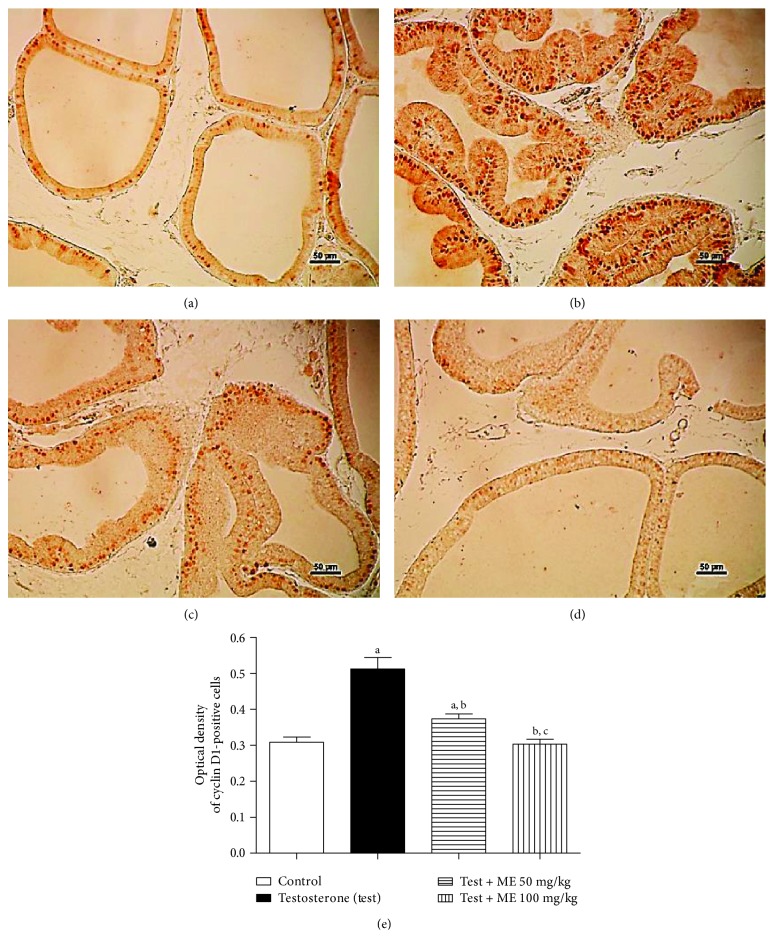
Immunohistochemistry photomicrographs of prostate sections showing the influence of 2ME on testosterone-induced hyperplasia of prostate expression of cyclin D1. (a) Section taken from control rats. (b) Section taken from the prostate of testosterone-treated group. (c) Section taken from the ventral prostate of the testosterone group cotreated with 50 mg/kg 2ME. (d) Section taken from the ventral prostates of testosterone groups cotreated with 100 mg/kg 2ME. (e) A graph showing the effect of 2ME cotreatment on prostate expression of cyclin D1. Scale bar (50 *μ*m). Data are expressed as mean ± SD (*n* = 6). a or b: statistically significant from control or testosterone group, respectively, at *P* < 0.05 using one-way ANOVA followed by Tukey's post hoc test.

The proliferation marker cyclin D1 protein expression was assessed immunohistochemically and showed moderate number of stained cells in the control group (Figure 2(a)). However, in the group which received testosterone, an increment of stained cells was noted, suggesting an elevation of the proliferation rate (Figure 2(b)). Cotreatment with 2ME was capable of significantly lowering the number of cyclin D1-positive cells, in comparison to the testosterone-treated group (Figures 2(c) and 2(d)). Cyclin D1-positive cells were quantified densitometrically. The data in Figure 2(e) indicated that 2ME (50 and 100 mg/kg) decreased cyclin D1 expression by 26 and 39%, respectively.

#### 3.4.2. Assessment of Bax and Bcl-2 mRNA Expression

**Figure 3:**
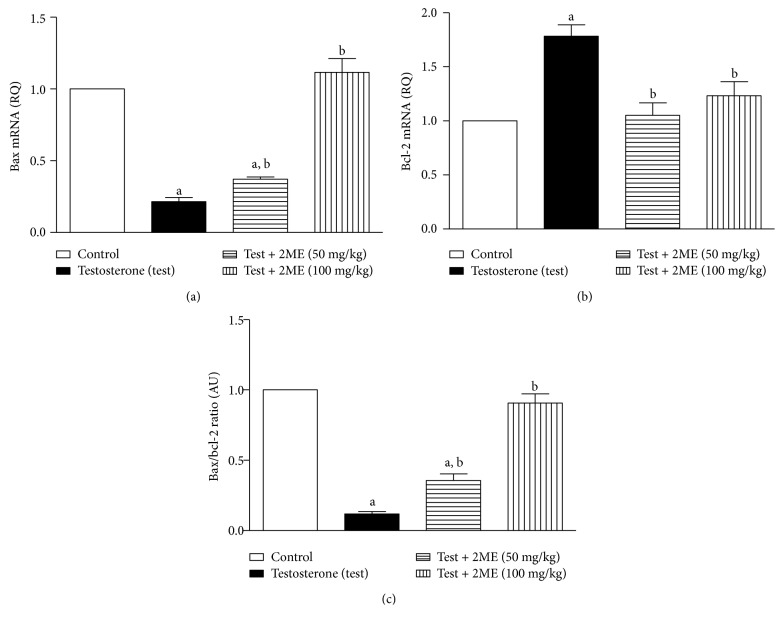
Effects of 2ME treatment on prostatic mRNA levels of Bax (a) and Bcl-2 (b) expressed as relative quantification (RQ) compared to the control group. (c) Bax/Bcl-2 ratio. Data are presented as the mean ± SD (*n* = 3). a or b: significantly different from control or testosterone-treated groups, respectively, at *p* < 0.05 using one-way ANOVA followed by Tukey's post hoc test.

The data in Figure 3 showed that testosterone enanthate significantly reduced Bax/Bcl-2 ratio by 83.5% relative to the controls. However, 2ME (50 and 100 m/kg) significantly protected against testosterone-induced reduction of mRNA expression Bax/Bcl-2 ratio by values amounting to 36 and 90% of the control value, respectively.

### 3.5. Evaluation of Prostate Content of IL-1*β*, TNF-*α*, NF-*κ*B (p65), and TGF-*β*1

**Figure 4:**
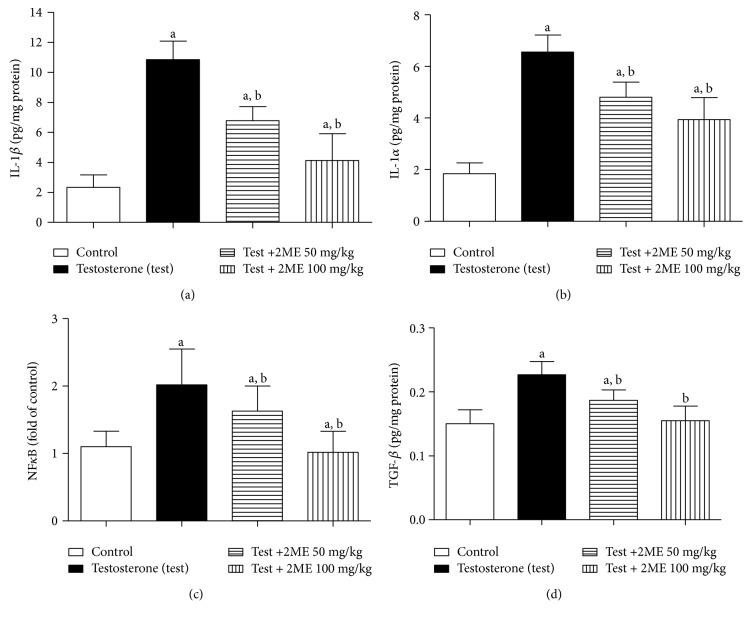
Effect of 2ME on prostate content of IL-1*β* (a), TNF-*α* (b), NF-*κ*B (c), and TGF-*β* (d) in testosterone-treated rats. Data are expressed as mean ± SD (*n* = 3). a or b: statistically significant from control or testosterone group, respectively, at *P* < 0.05 using one-way ANOVA followed by Tukey's post hoc test.

IL-1*β*, TNF-*α*, NF-*κ*B (p65), and TGF-*β*1 levels were significantly higher in the prostate tissues of testosterone-treated animals compared to controls. Treatment with 2ME (50 and 100 mg/kg) significantly lowered IL-1*β* by 38 and 62%, TNF-*α* by 27 and 40%, NF-*κ*B (p65) by 19 and 50%, and TGF-*β*1 by 18 and 32%. There was no statistical difference on the tissue levels of TGF-*β*1 between animals receiving 100 mg/kg 2ME and control group (Figures 4(a)–4(d)).

### 3.6. Assessment of HIF-1*α* and Phospho-Smad2

**Figure 5:**
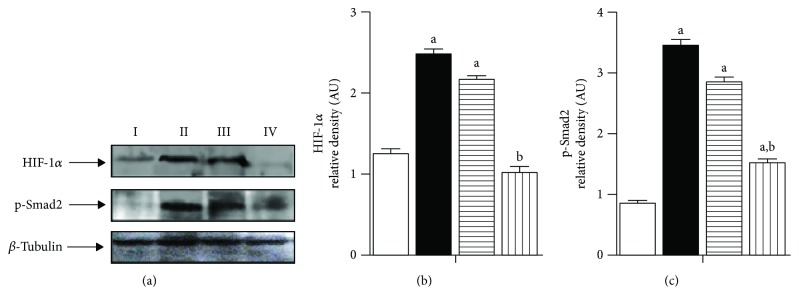
(a) Western blot analysis of HIF-1*α* and p-Smad2 expression in prostate tissues. I: untreated control; II: testosterone-induced BPH group (3 mg/kg, 5 days/week for 2 weeks, S.C.); III: testosterone-induced BPH treated with 2ME (50 mg/kg); IV: testosterone-induced BPH treated with 2ME (100 mg/kg). (b) Densitometric quantitation of HIF-1*α*. (c) Densitometric quantitation of p-Smad2. Data are expressed as mean ± SD (*n* = 3). a or b: statistically significant from control or testosterone group, respectively, at *P* < 0.05 using one-way ANOVA followed by Tukey's post hoc test.

The expression levels of HIF-1*α* in prostate tissue homogenates were investigated by Western blot (Figure 5). Testosterone increased HIF-1*α* and p-Smad2 protein expression in comparison to control rats. 2-Methoxyestradiol (50 and 100 mg/kg) significantly lowered protein expression of HIF-1*α* by 12% and 60% and p-Smad2 by 18 and 56% (Figure 5).

## 4. Discussion

In males, BPH is the most widespread benign tumor exhibiting an age-related incidence [21]. Imbalance between cell proliferation and cell apoptosis, oxidative stress, and inflammation are the key players in BPH development [22–24]. The annoying effects of BPH negatively affect men's quality of life. The main pharmacological approaches for the management of BPH embrace the use of 5-*α* reductase and adrenergic *α*-1 inhibitors [25]. However, these are associated with a variety of side effects [26, 27]. 2-Methoxyestradiol is the end metabolite of estrogens. It is produced by the oxidation of 17*β*-estradiol by the enzyme, cytochrome P450, and subsequently by the O-methylation by catechol-o-methyl transferase (COMT) (5). COMT gene polymorphism has been reported to associate with BPH [28]. 2-Methoxyestradiol has antiproliferative [6], antioxidant [29], and anti-inflammatory [30] properties. Our goal was to explore the ability of 2ME to inhibit the testosterone-induced BPH in rats.

Treatment of animals with 2ME (50 and 100 mg/kg) for 2 weeks ameliorated testosterone-induced increases in prostate weight and index. Further, histopathological investigation confirmed these findings as 2ME almost preserved the normal histological architecture of prostatic tissue and statistically prevented the increase in glandular epithelial height. This is in accordance with the known antiproliferative properties of 2ME [31] that made it a very promising candidate drug in phase II study in patients with castrate-resistant prostate cancer [10]. Many reports highlighted a role for oxidative stress in testosterone-induced BPH [32]. Our data indicate that 2ME exhibited antioxidant properties as it ameliorated the increase in prostate content of lipid peroxides and the exhaustion of SOD. However, values of GSH content and CAT activity were not significantly altered. This may be due to limitations of the analytical methods used. These findings are in accordance with many reports that attributed the preventive effects of several agents to their antioxidants properties [33–35].

Testosterone treatment resulted in disturbing the balance between prostatic proliferation and apoptosis in favor for proliferation and cell cycle advancement. This was evidenced by the decrease in Bax/Bcl2 ratio and increase in cyclin D1 expression. This is in line with our previous study on chrysin against testosterone-induced BPH [36]. 2-Methoxyestradiol in both dose levels significantly enhanced Bax/Bcl2 ratio and mitigated the increase in cyclin D1 expression. This is supported by the notion that 2ME enhances Bax and inhibits Bcl2 expression in prostate and other cell lines [37–39]. Also, the ability of 2ME to inhibit cyclin-D1 in different cell types has been previously reported [40, 41].

Prostatic inflammation represents an important factor in influencing the development of BPH [42]. In BPH patients, prostate volume and symptom score were significantly higher in patients with high-grade prostatic inflammation [43]. Experimentally, several reports indicated that testosterone-induced BPH is mediated by inflammation and protective agents were shown to have anti-inflammatory properties [44, 45]. In the current study, testosterone significantly elevated prostatic content of IL-1*β* and TNF-*α*. However, 2ME at both doses decreased such inflammatory mediators. This concurs with reports demonstrating the ability of 2ME to inhibit IL-1*β* and TNF-*α* expression in renal tissues [46]. Also, our data indicate that testosterone-mediated inflammation and hyperplasia are mediated by elevated NF-*κ*B and TGF-*β* signalling. This gives an additional explanation for the observed decrease in prostatic Bax/Bcl2 ratio [47].

The protective effects of 2ME against the rise in NF-*κ*B can be also linked to its antioxidant activity as oxidative stress can also persuade inflammatory responses via redox-sensitive transcription factor activation including NF-*κ*B [48]. NF-*κ*B is a transcription factor consisting of p65 and p50 subunits that is retained within the cytoplasm in an inactive form. Upon activation, it is transported into the nucleus [49] and controls the expression of several proinflammatory mediators as TNF-*α* [50]. This justifies the detected elevation of IL-1*β* and TNF-*α* in testosterone-treated animals and is in line with their known role in promoting prostate cell proliferation [51]. Our results gain support by the previous findings that 2ME causes functional repression of TGF-*β* signaling in uterine fibroid cells [52].

The ability of 2ME to target TGF-*β* in prostatic tissues was further explored by assessing upstream HIF-1*α* and the downstream p-Smad2. In the present work, testosterone significantly enhanced HIF-1*α* protein expression in rat prostates. This is consistent with previous findings that tissue hypoxia and HIF-1*α* are implicated in testosterone-stimulated growth of the rat prostate [53]. On the other hand, 2ME significantly hampered HIF-1*α* expression when compared to testosterone alone-treated animals. It has been previously reported that the antiproliferative activity of 2ME involves inhibition of HIF-1*α* [54, 55]. In addition, inhibition of HIF-1*α* has been reported to inhibit inflammatory cytokines and TNF-*α* [56, 57]. Thus, the pathogenic role of HIF-1*α* in prostatic hyperplasia gains extra support by the observed elevation of TNF-*α* and IL-1*β* by testosterone [58].

**Figure 6:**
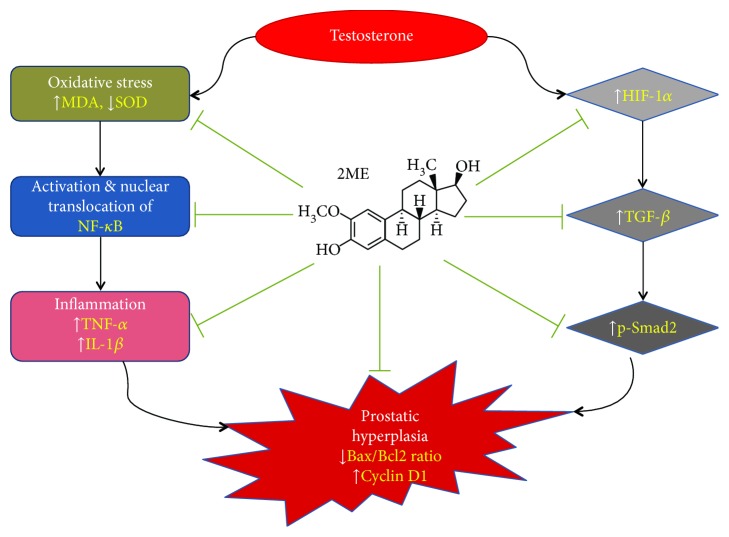
Schematic presentation of the proposed mechanisms of the 2ME protective effects against testosterone-induced prostatic hyperplasia in rats.

The proposition that 2ME beneficial effects are mediated by inhibiting HIF-1*α*-TGF-*β* was confirmed by the finding that p-Smad2 protein expression was significantly decreased in the treated animals. This finding is in accordance with the notion that Smad proteins are implicated in the cellular response to hypoxia [59]. Also, inhibition of TGF-*β*-Smad signaling was reported to decrease prostate cancer cell proliferation [60]. Besides, our data are supported by the reported ability of 2ME to inhibit TGF-*β*-Smad in uterine fibroid cells [52]. Our suggested mechanisms of the 2ME protection against testosterone-induced hyperplasia are summarized in Figure 6. In brief, 2ME exerts antioxidant activity that leads to inhibition of NF-*κ*B activation and nuclear translocation, which eventually leads to decreased release of inflammatory mediators. Further, 2ME inhibits HIF-1*α* expression, which would lead to decreased expression of TGF-*β* and p-Smad2. Both arms end up with decreased prostatic cell proliferation.

## 5. Conclusion

2-Methoxyestradiol protects against testosterone-induced PBH in rats in a dose-related manner as the higher dose (100 mg/kg) showed better preventive effects. This can be attributed to, at least partly, inhibition of HIF-1*α*/TGF-*β*/Smad2 axis.

## Figures and Tables

**Table 1:** Nucleotide sequence of primers used for gene expression of Bax, Bcl2, and B2m.

Gene	Forward	Reverse
Bax	AGA GGA TGG CTG GGG AGA CAC	TCC ACA TCA GCA ATC ATC CTC TG
Bcl-2	TCT TTG AGT TCG GTG GGG TCA	GTT CCA CAA AGG CAT CCC AGC
B2m	GAT GTC AGA TCT GTC CTT CAG CA	GTC TCG GTC CCA GGT GAC G

**Table 2:** Effect of 2-methoxyestradiol (2ME) on prostate weight and prostate index (prostate weight/body weight ratio) in testosterone-induced BPH in rats.

	Rat weight (g)	Prostate weight (mg)	Prostate index (×10^3^)
Control	283 ± 42.0	0.471 ± 0.048	1.54 ± 0.063
Testosterone (test)	295 ± 18.9	0.830^a^ ± 0.058	2.97^a^ ± 0.326
Test + 2ME 50 mg/kg	233^a,b^ ± 13.6	0.552^a,b^ ± 0.067	2.45^a,b^ ± 0.271
Test + 2ME 100 mg/kg	235^a,b^ ± 17.8	0.397^a,b^ ± 0.051	1.57^b^ ± 0.162

Data are expressed as mean ± SD. a or b: statistically significant from control or testosterone group, respectively, at *P* < 0.05 using one-way ANOVA followed by Tukey's post hoc test.

**Table 3:** Effect of 2-methoxyestradiol (2ME) on MDA and GSH content and SOD and CAT activities in prostate tissues of testosterone-induced BPH in rats.

	MDA (nmol/mg protein)	GSH (mmol/mg protein)	SOD (U/mg protein)	CAT (U/mg protein)
Control	0.259 ± 0.043	0.158 ± 0.008	40.6 ± 2.40	0.56 ± 0.034
Testosterone (test)	1.05^a^ ± 0.164	0.164 ± 0.004	28.2^a^ ± 2.56	0.52 ± 0.038
Test + 2ME 50 mg/kg	0.726^a,b^ ± 0.077	0.156 ± 0.004	33.7^a,b^ ± 1.65	0.52 ± 0.045
Test + 2ME 100 mg/kg	0.439^a,b^ ± 0.66	0.155 ± 0.002	38.4^b^ ± 1.22	0.54 ± 0.051

Data are expressed as mean ± SD. a or b: statistically significant from control or testosterone group, respectively, at *P* < 0.05 using one-way ANOVA followed by Tukey's post hoc test.

## Data Availability

The data used to support the findings of this study are available from the corresponding author upon request.
